# New Mechanisms for Regulation of Intracellular Collagen Degradation in Fibrotic Lesions of Periodontal Tissues

**DOI:** 10.1096/fj.202502454R

**Published:** 2025-10-26

**Authors:** Wing Hei Wong, Ralph A. Zirngibl, Morris F. Manolson, Christopher A. McCulloch

**Affiliations:** ^1^ Faculty of Dentistry University of Toronto Toronto Ontario Canada

**Keywords:** extracellular matrix, lysosomes, Rabs, trafficking

## Abstract

Fibrosis is involved in the pathogenesis of 45% of deaths in developed countries. Thus, defining the signaling systems that promote fibrotic lesions is important for developing therapies that can preserve human health. Fibrosis affects several organs and is also commonly manifest in the gingival connective tissue of tooth‐supporting tissues, which exhibit very rapid turnover of collagen. Gingiva, therefore, provides an instructive model system for determining how the signals that regulate the loss of the balance of collagen synthesis and degradation are dysregulated in gingival tissue enlargement, a common fibrotic lesion that is associated with considerable morbidity. Notably, these fibrotic lesions affect a high proportion of patients who are treated with drugs that affect Ca^2+^ signaling and trafficking of collagen through vacuolar compartments. As Ca^2+^ signaling is functionally related to the control of vacuolar trafficking by the small G‐proteins known as Rabs, here we discuss dysregulation of collagen degradation in the gingiva with a particular focus on signaling through Rabs and on the role of vacuolar ATPases in lysosomal acidification to optimize collagen degradation. We consider how exploration of Rabs‐mediated regulation of the intracellular degradation pathway may inform new approaches for the clinical management of fibrotic lesions.

## Graphical abstract

Drug‐induced gingival overgrowth involves dysregulation of intracellular collagen degradation. The mechanisms that are involved in the regulation of lysosomal collagenolytic activity are poorly understood. Here, we focus on how cathepsins, Rabs, and V‐ATPases regulate intracellular collagen degradation and, in particular, the vesicular trafficking and lysosomal acidification that are required for optimization of collagenolytic activity.

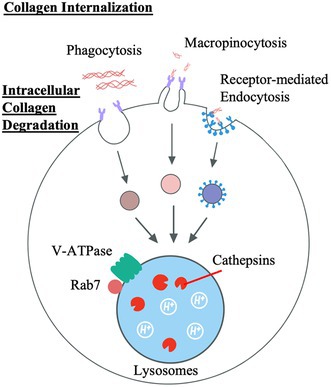

## 1 Introduction

Fibrosis is involved in the pathogenesis of > 45% of deaths in developed countries [1] and is therefore of considerable interest in the clinical management and preservation of human health. In organs such as the lung, liver, and heart, fibrosis can spread within the organ, thereby contributing to organ failure and, in advanced progressive diseases, death [2]. One of the tissues that is commonly affected by fibrosis is the gingival fiber system of the periodontium [3], which is involved in tooth attachment and support. Periodontal tissues and gingiva, in particular, exhibit rapid and tightly balanced synthesis and degradation of collagen to preserve fiber structure, tissue homeostasis, and function [4]. As a result of this rapid collagen turnover (Box 1), collagen‐producing and degrading cells in these tissues (i.e., gingival and periodontal ligament fibroblasts) provide useful models for identifying critical regulatory systems that preserve collagen homeostasis, particularly those systems that modulate collagen degradation.

BOX 1 **Role of collagen remodeling in preservation of oral health.**

Type I collagen is the major protein of the organic phase of oral connective tissues, including alveolar bone, dentin, cementum, and periodontal ligament [5]. Collagen remodeling is required for the success of various dental treatments, including orthodontic tooth movement [6]. Dysregulated collagen remodeling affects gingiva and bone in periodontitis, which manifests as loss of alveolar bone and gingival connective tissues [7]. In contrast, excessive collagen synthesis occurs in oral submucous fibrosis, a lesion that can undergo malignant transformation [8]. Dysregulation of ECM turnover induces the formation of a stiff matrix, which further potentiates fibrosis [9] and is seen in gingival enlargement.

Small perturbations in the balance of collagen synthesis and degradation are rapidly manifest as altered morphology, structure, and function of gingival tissues. These alterations are widely recognized in two high‐prevalence infections of the periodontium: gingivitis and periodontitis. In these diseases, dysregulation of gingival collagen homeostasis can be caused by disturbances in the rates of collagen synthesis and degradation. These disturbances lead to a net increase of disorganized collagen within the tissue, which contributes to the formation of gingival fibrosis and is commonly observed in drug‐induced gingival enlargement [10, 11, 12, 13]. Notably, the American Academy of Periodontology, in their consideration of nomenclature for periodontal diseases, has also suggested the use of the term “drug‐influenced gingival enlargement” for these conditions. In this review, we consider the etiology of fibrosis in chronic inflammatory lesions and drug‐induced lesions of the gingiva. Although previous reports [14, 15, 16, 17, 18, 19] described how disturbances in the rates of collagen synthesis and extracellular degradation contribute to gingival fibrosis, here we consider the control systems that regulate intracellular collagen degradation to provide new insights into how gingival fibrosis develops.

## 2 What Is Fibrosis?

Fibrosis involves the pathological replacement of normal tissues by an expanded, disorganized, and collagen‐rich extracellular matrix (ECM) in affected tissues and organs. The formation of fibrotic lesions in affected tissues is a common sequela of many chronic inflammatory diseases, invasive cancers, and dysregulated wound healing processes. In response to chronic inflammation or after injury, a wound healing response is initiated, which features increased expression of a large repertoire of soluble mediators and signaling systems that include the expression of pro‐fibrotic cytokines, mediators of oxidative stress, and activation of matrix adhesion molecules. If the wound healing response is prolonged and poorly regulated, excessive formation of collagen and abnormal scar tissue may form [2], which contributes to tissue dysfunction and, over the long term, may lead to overt organ failure [20]. Further, alterations in the molecular repertoire of the fibrotic ECM can provide signals that promote a positive feedback loop, which compounds the progression of fibrosis [20]. The major ECM constituents of fibrotic lesions are collagens [20], which are the most abundant proteins in mammals [21] and are particularly enriched in many soft connective tissues [22], including gingiva.

## 3 Gingival Enlargement

Fibrosis is a common feature of gingival inflammatory lesions, which typically exhibit elevated numbers of epithelial cells and fibroblasts [23], increased abundance of matrix proteins in the lamina propria, expansion of normal gingival dimensions, and distortions of overall tissue morphology [18]. These latter alterations are cardinal features of gingival enlargement, which is often associated with poor oral hygiene [10]. In the absence of effective biofilm control, after surgical resection of excess tissue, gingival enlargement commonly recurs (Box 2) [10, 24].

BOX 2 **Gingival enlargement.**

Gingival enlargement is caused by imbalances of ECM turnover [10], which can be induced by biofilms and by a variety of drugs including immunosuppressants, anticonvulsants, and calcium channel blockers [25]. Gingival enlargement presents as tissue expansion, because, in part, of the accumulation of high‐abundance ECM components like collagen [10]. In most cases, enlargement must be removed surgically to maintain oral health.

## 4 Etiology of Enlargement in Periodontal Diseases

Two of the highest prevalence infectious diseases of humans are associated with gingival enlargement. First, gingivitis is a reversible, inflammatory disease of gingival tissues that affects > 80% of the global human population [26] and is caused by anaerobic biofilms [27]. Second, periodontitis is a chronic, inflammatory disease of the periodontium, also driven by biofilms that cause irreversible destruction of alveolar bone and soft tissues. Periodontitis affects ~30% of adults in the western countries (e.g., the United States) [28]. In both of these diseases, biofilm‐driven inflammation can promote gingival fibrosis [2] in a subpopulation of patients [29]. Enlargement is also associated with the epithelial to mesenchymal transition [24], which can increase the density of fibroblasts in fibrotic lesions.

In many different organs and tissues with chronic inflammation, tissue‐resident fibroblasts are activated by locally produced cytokines and growth factors. Similarly, in the initial phases of gingivitis in response to tooth‐adherent, anaerobic biofilms, there is rapid recruitment of immune and endothelial cells, which express cytokines that mediate the inflammatory responses [30]. In a sub‐population of patients with chronic gingival inflammation, gingivitis can convert to periodontitis (the advanced lesion), which is associated with irreversible, net destruction of periodontal attachment [30] as well as isolated deposition of disorganized collagen that is often spatially separate from the inflammatory focus [31]. In addition to inflammation, gingival enlargement can be driven by iatrogenic dental treatments [32] and smoking, which can also promote increased expression of type I collagen by gingival fibroblasts [33, 34].

## 5 Pathogenesis of Drug‐Induced Gingival Enlargement

In addition to the localized accumulation of gingival collagen seen in gingivitis and periodontitis, certain drugs (e.g., Cyclosporin, Nifedipine, and Dilantin) [35] are associated with the overt formation of gingival enlargement in subpopulations of patients (Table 1).

**TABLE 1:** Drugs that promote gingival enlargement.

Drug type		Mechanism of action	Effect on gingival enlargement (+++ high incidence, ++ moderate incidence, + low incidence)	Effect on other fibrotic systems (+ induces fibrosis, − reduces fibrosis)
Immunosuppressant	Cyclosporin A	Forms a complex with cyclophilin D and inhibits calcium release [36]	++ [18, 37]	+/− [38, 39, 40]
	Tacrolimus	Interacts with intracellular binding proteins and forms a complex with calcineurin [41]. Inhibits calcineurin phosphatase and calcium‐dependent events [42].	++ [43]	+/− [44, 45, 46]
Anti‐hypertensives	Dihydropyridines (Nifedipine, Amlodipine, Felodipine)	Inhibits extracellular calcium influx via L‐type calcium channels [47]	++ [18, 48, 49]	− [50]
	Non‐dihydropyridines (Verapamil)		+ [18, 30]	− [51]
Anti‐convulsant	Dilantin/phenytoin	Inhibition of calcium uptake [52] and fibroblast‐mediated collagen internalization [16, 53]	+++ [18, 49]	+ [54]
	Phenylbarbitone	Inhibition of calcium uptake [52]	+++ [55]	+ [56, 57]

Several classes of drugs are associated with increased incidence of gingival enlargement, which include immunosuppressant drugs used for the treatment of organ transplant rejection (70% increase in prevalence of enlargement), anti‐hypertensives (30% increase), and epileptic seizure control drugs (65% increase) [58]. All of these drugs dysregulate Ca^2+^ signaling, which is associated with integrin‐mediated phagocytosis of collagen [36]. Ca^2+^ signaling may also impact the trafficking of collagen through vacuolar compartments that are critical for intracellular collagen degradation. It is not known whether the pathogenesis of gingival enlargement induced by inflammation is the same as drug‐induced enlargement, but both types of lesions are associated with dysfunction of collagen regulatory mechanisms [10].

Immunosuppressants are used after transplant surgery to reduce rejection of the newly transplanted organ [10]. Cyclosporin A and Tacrolimus are examples of immunosuppressants that are associated with gingival enlargement and with hyperplasia of gingival epithelial cell populations [59]. Cyclosporin A binds to cyclophilin D, which promotes its detachment from the mitochondrial permeability transition pore to inhibit the release of Ca^2+^. Cyclosporin A also downregulates β1 integrin binding to collagen, a critical initial step for collagen degradation by phagocytosis [36].

Many commonly used anti‐hypertensives (e.g., Nifedipine) [10] are Ca^2+^ channel blockers that inhibit voltage‐dependent L‐type Ca^2+^ channels [60, 61, 62, 63] in the myocardium and vascular smooth muscle [64]. These drugs include dihydropyridines (e.g., Nifedipine, Amlodipine, Felodipine) or non‐dihydropyridines (e.g., Verapamil) [64, 65]. Ca^2+^ channel blockers can alter the activation of collagenases in cultured cardiac fibroblasts [66]. This interference may contribute to the collagen build‐up in the gingiva of patients taking amlodipine [19], a drug that blocks integrin‐dependent collagen phagocytosis in rat gingiva [15].

Diphenylhydantoin (Dilantin) and phenobarbitone are commonly used for the clinical management of epileptic seizures [10]. Dilantin contributes to gingival enlargement [17] by inhibiting Ca^2+^ signaling, integrin‐mediated collagen internalization by fibroblasts [16, 53] and by other, not as well‐characterized mechanisms.

In general, although none of the drug classes described above promote marked increases in collagen synthesis, they are strongly associated with the inhibition of collagen degradation, which is central to the pathogenesis of drug‐induced gingival enlargement [15, 36]. Although these various classes of drugs exhibit quite different pharmacological mechanisms, they all affect Ca^2+^ signaling. As a second messenger, Ca^2+^ influences early, adhesion‐dependent processes related to collagen internalization [67].

## 6 Intrinsic Features of Collagen Structure That Enable Fibrosis

The most abundant protein in mammals and of the ECM is collagen [68]. As a result, tight regulation of the balance between collagen synthesis and degradation is important for preservation of ECM homeostasis and ultimately, human health. Disturbances of collagen homeostasis contribute directly to the development of fibrotic lesions. Collagen molecules are comprised of amino acid chains with a repeating primary sequence of Glycine‐Proline‐X or Glycine‐X‐Hydroxyproline. The very high abundance (1/3) of the small amino acid glycine in collagen allows tight packing of the triple helix, a property that facilitates the assembly of polypeptide chains with the high tensile strength that characterizes collagen fibers [69].

The structure of collagen contributes to its insolubility and resistance to most proteases. Only a few enzymes can degrade cross‐linked collagen fibers. In the extracellular environment, the kinetics of collagen cleavage are slow because of limited access to the scissile bonds [70]. As a result, when excessive fibrillar collagen is deposited in fibrotic lesions, remodeling of this heavily cross‐linked collagen is very slow, and the function of the original tissue or organ is often compromised by the stiffness of the interstitium. The formation of fibrotic lesions in organs and tissues is, in the first instance, reliant on the formation and deposition of extracellular collagen, a process that we describe below.

## 7 Collagen Synthesis

The synthesis of type I collagen, the most abundant type of collagen in mammalian connective tissues, including the periodontium [71], comprises three important steps: transcription, translation, and post‐translational modifications [69]. In the nucleus, pro‐α1 and pro‐α2 mRNA are transcribed, subsequently trafficked to the cytoplasm, and there translated into pre‐pro‐polypeptide chains [69]. Next, the pre‐pro‐polypeptide undergoes post‐translational modifications in which the three pro‐α‐chains are assembled into a triple‐helical conformation as procollagen [69], which traffics to the Golgi for further modification and supramolecular assembly. Because of the large size of procollagen molecules, secretion from the ER to the Golgi requires the involvement of specialized ER exit sites, which enable vesicular trafficking of large proteins like collagen to the Golgi. In these compartments, nascent collagen molecules are further modified by coat protein II and interactions with TANGO1 [72] to enable transport. After the exit of procollagen molecules from the Golgi, extracellular cleavage of telopeptides enables the assembly of collagen fibrils and cross‐linking [69], which are critical for the formation of functional collagen fibers with high tensile strength.

## 8 Intracellular Degradation of Defective Nascent Collagen

In the process of protein synthesis, errors in protein assembly, post‐translational modification, or folding can perturb endoplasmic reticulum (ER) homeostasis. The unfolded protein response preserves ER homeostasis by redirecting misfolded proteins for degradation by autophagy [73]. Misfolded proteins can be degraded by endosomal/lysosomal‐regulated micro‐ER‐phagy or by autophagosome‐regulated macro‐ER‐phagy [73]. In the context of matrix homeostasis, after collagen is synthesized, 20%–40% of newly generated procollagen is degraded rapidly by ER‐phagy [73, 74]. In this process, prolyl and lysyl residues of collagen act as markers for collagen degradation [75]. Quality control during collagen synthesis ensures that every third amino acid is glycine and that the presence of hydroxyprolyl residues can appropriately contribute to the formation and stability of triple helices [75]. Degradation of nascent collagen is not mediated by interstitial collagenases (as occurs in the extracellular pathway) or by the phagocytic intracellular pathway [74, 75]. Instead, degradation of misfolded intracellular collagen via ER‐phagy is considered a distinct process.

## 9 Extracellular Degradation of Collagen in the ECM

Preservation of the dentogingival junction is very dependent on the maintenance of optimal collagen fiber structure [76], a process that requires collagen remodeling. As discussed above, the tightly wound helical conformation of collagen molecules and its assembly into highly cross‐linked fibers render it susceptible to only a few cysteine proteases (e.g., cathepsins) and to certain matrix metalloproteinases (MMPs) that act as interstitial collagenases [77]. As noted above, collagen degradation is of considerable import for homeostatic regulation of the ECM, particularly for the periodontium because of the rapid synthesis and turnover of collagen that is manifest in periodontal tissues [4]. Physiological degradation of extracellular collagen (i.e., previously synthesized and exported) can involve the extracellular and intracellular pathways (Box 3).

BOX 3 **Collagen degradation pathways involved in matrix remodeling.**

Collagen homeostasis is critical for maintaining healthy tissues. Degradation of collagen in the ECM involves the extracellular and intracellular pathways [76]. MMPs cleave and degrade collagen in the extracellular degradation pathway [78]. In the intracellular pathway, partially cleaved and intact collagen can be internalized and degraded via cathepsins. Although the extracellular pathway has been studied in considerable depth, the regulators of the intracellular pathway for collagen degradation are not well defined [77].

The intracellular and extracellular pathways of collagen degradation use discrete pathways with separate types of proteases that can mediate proteolysis in these very different environments (Box 4).

BOX 4 **Extracellular and intracellular collagenases.**

Enzymes with collagen‐degrading activity contribute to the regulation of ECM homeostasis [79]. There are two general types of collagenases: those that act in the extracellular space (e.g., MMPs) and those that act primarily in intracellular vacuolar compartments (i.e., cathepsins) [80]. MMPs are zinc‐dependent endopeptidases that cleave collagen at neutral pH, as found in the extracellular space [79, 81]. Conversely, cathepsins, which are cysteine proteases, must undergo cleavage in acidic environments for activation and performance of collagen degradation [79].

The extracellular collagenolytic pathway largely involves the activity of Zn^2+^‐dependent endoproteinases (i.e., MMPs), some of which can act as interstitial collagenases and exhibit triple helicase activity [82]. These enzymes include MMP‐1, MMP‐2, MMP‐8, MMP‐13, MT1‐MMP, and MT3‐MMP [77]. Different MMPs preferentially cleave specific types of collagens: type I and III collagens are cleaved by MMP‐1 and MMP‐8, whereas type II collagen in cartilage is preferentially cleaved by MMP‐13 [77]. Collagen degradation mediated by interstitial collagenases generates ¾ and ¼ collagen fragments, a process that is reliant on catalytic attack of the collagen molecule between Gly775 and Ile776 of the alpha 1 (I) chain. This thermodynamically favorable site for cleavage is highly conserved and is essential for the degradation of type I collagen by collagenases [77]. In contrast with the extracellular collagen degradative activity of MMPs, cathepsin‐mediated collagenolysis is mainly restricted to the intracellular pathway, although cathepsin K can also degrade extracellular collagen under specific conditions [83].

## 10 First Step of Intracellular Degradation: Collagen‐Binding Receptors

The intracellular collagen degradation pathway begins with the binding of different types of collagen by specific adhesion receptors [84] (Table 2).

**TABLE 2:** Collagen binding receptors.

Type of collagen binding receptor		Type of collagen
Integrins	α1β1 and α10β1	Collagens IV and VI [85]
	α2β1 and α11β1	Fibrillar collagens [85]
Discoiding domain receptors (DDRs)	DDR1	Collagens I–VI and VIII [86]
	DDR2	Fibrillar collagens I and III [86]
Glycoprotein VI (GPVI)		Fibrillar collagen [87]
LAIR‐1 (leukocyte‐associated immunoglobulin‐like receptor 1)		Transmembrane and extracellular matrix collagens [88]
Mannose receptor (CD206)		Collagen types I, II, III, IV, and gelatin/denatured collagen [89]
Urokinase plasminogen activator receptor protein (UPARAP/Endo180)		Collagen I, IV, and V [84, 90]

One of the most deeply studied families of collagen binding receptors is the integrins, which bind to specific GFOGER motifs in the collagen molecule [85]. The α1β1 and α10β1 integrins bind to collagen types IV and VI, whereas α2β1 and α11β1 integrins bind to fibrillar collagens [85]. The fibrillar collagens are particularly important in the generation of fibrotic lesions and include Types I (most abundant and broadly distributed), II (cartilage), III (skin, blood vessels, organs), V (placenta, cell surfaces), XI (cartilage), XXIV, and XXVII [91].

A large body of data indicates that direct interactions of collagen binding integrins with specific amino acid sequences in collagen (Glycine‐Phenylalanine‐Hydroxyproline‐Glycine‐Glutamic Acid‐Arginine) mediate cell adhesion. Notably, in cartilage, cell adhesion to collagen involves the binding of integrin‐containing adhesion suprastructures to non‐collagenous molecules in the fibril periphery; adhesion is not apparently mediated by the fibrillar cores of collagen [92]. In addition to mediating cell adhesion to collagen, specific collagen‐binding integrins can also induce MMP expression: α_2_β_1_ integrins promote MMP‐1 and MMP‐2 activation and increase collagenolysis [93].

In the general context of intracellular collagen degradation, the activation of collagen‐binding β1 integrins likely governs early, rate‐limiting steps for internalization [93] and thus dysregulation of the function of collagen‐binding integrins may be important in the formation of fibrotic lesions. Although the fibrillar collagen‐binding integrins (i.e., those containing a β1 integrin subunit) are important for collagen internalization and matrix homeostasis, αv integrins (αvβ1, αvβ3, αvβ5, αvβ6, and αvβ8) contribute to fibrosis [93], in part by activating the pro‐fibrotic growth factor, TGF‐β.

Another family of fibrillar collagen receptors, the discoidin domain receptors (DDRs), is collagen‐activated tyrosine kinases that bind to a GVMGFO motif in collagen, which facilitates DDR activation [86, 94]. There are two types of DDRs, DDR1 and DDR2. DDR1 is mainly expressed by epithelial cells and binds to collagens I–VI and to VIII. DDR2 is expressed mainly by mesenchymal cells and binds to the fibrillar collagens I and III [86]. DDRs are implicated in the formation of fibrotic lesions [95] but there is only limited evidence that they regulate collagen internalization [96]. Glycoprotein VI (GPVI) and leukocyte‐associated immunoglobulin‐like receptor 1 (LAIR‐1) bind to collagen and enable platelet activation and immune responses, respectively [88, 94] but similar to the DDRs, their direct involvement in collagen internalization is very limited. The mannose receptor (CD206), expressed by immune cells, binds to collagens I–IV and denatured collagen in order to facilitate immune responses and the endocytosis of collagen [89]. The Urokinase Plasminogen Activator Receptor Protein (UPARAP/Endo180), which is expressed in mesenchymal cells, is involved in the turnover of ECM components and collagen V and exhibits somewhat lower binding affinity for collagens I and IV [84, 90]. UPARAP/Endo180 is the principal binding receptor for collagen internalization in the receptor‐mediated endocytic intracellular degradation pathway [97].

## 11 Collagen Internalization Pathways

**FIGURE 1:**
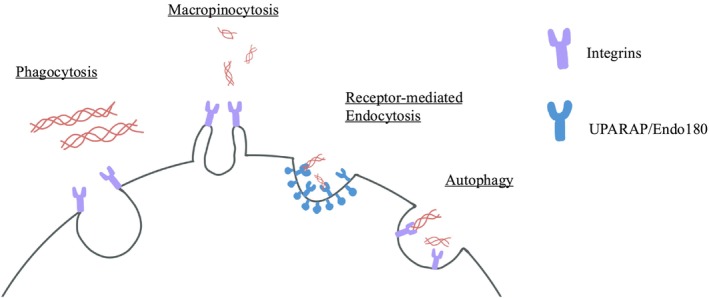
Internalization of collagen through various intracellular degradation pathways.

Following collagen binding, discrete pathways and collagen receptors enable the recognition and internalization of soluble collagen molecules, partially degraded fibrils, or intact fibrils (Figure 1). These pathways include receptor‐mediated endocytosis, micropinocytosis, and collagen phagocytosis [82].

### 11.1 Receptor‐Mediated Endocytosis

This pathway involves endocytosis of soluble collagen generated as a result of MMPs and other extracellular proteases, and is mediated by binding through the urokinase plasminogen activator receptor‐associated protein (uPARAP/Endo180) and collagen internalization into clathrin‐coated endocytic vesicles [77].

### 11.2 Macropinocytosis

Collagen fragments partially degraded by membrane‐anchored interstitial collagenases (e.g., MT‐1) are internalized by macropinocytosis [77]. In this process, soluble, pre‐cleaved collagen fragments enter macropinosomes [98]. Macropinocytosis of collagen is prominent in inflammatory sites [99].

### 11.3 Phagocytosis

The internalization of insoluble, relatively large (> 0.5 μm in length) collagen fibrils via integrins [83, 100] involves an actin‐mediated internalization and degradation process. The partially degraded fibrils are engulfed into phagosomes [100] prior to digestion in phagolysosomes. Receptor‐mediated endocytosis, micropinocytosis, and phagocytosis converge at the point of lysosomal fusion with phagosomes.

## 12 Intracellular Collagen Degradation

In contrast with MMPs, the degradation of collagen in the intracellular pathway [82] occurs in lysosomes, a process that is mediated by cathepsins and other cysteine proteases. Cathepsins, which are synthesized as a pro‐peptide, degrade protein cargoes in lysosomes [79]. Notably, cathepsins in general and cathepsin L and K in particular, when located in acidic compartments, are much more efficient collagenases than interstitial collagenases of the MMP family [101]. Therefore, consideration of how their catalytic activity is regulated relates directly to the function of the phagocytic pathway in collagen degradation. Cathepsins hydrolyze peptide bonds using a mechanism that involves a nucleophilic cysteine residue, which subsequently forms a thioester intermediate with the substrate.

**FIGURE 2:**
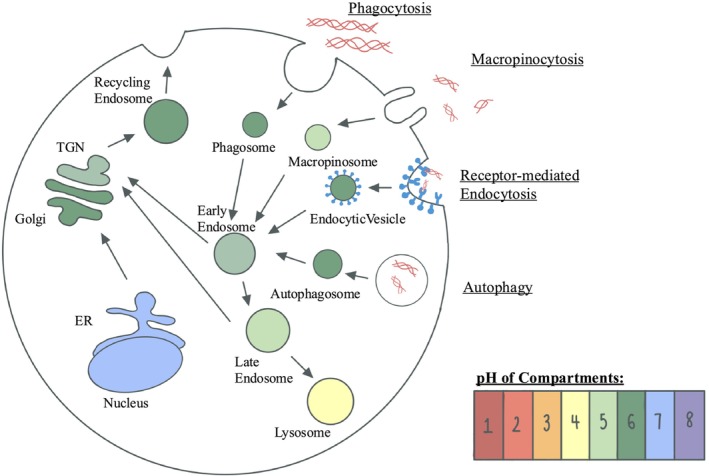
Overview of the pH of various vacuolar compartments in the intracellular collagen degradation pathway.

As internalized collagen traverses the degradation pathway, the compartments exhibit increased acidity approaching lysosomes [102, 103, 104, 105, 106, 107]. The acidic pH of lysosomes enhances the efficiency of cathepsin‐mediated collagen degradation (Figure 2) [79]. The regulation of lysosomal pH may play a critical role in optimizing intracellular collagen degradation.

## 13 Cathepsins

Cathepsins are of central importance in ECM remodeling. For example, interference with the expression of cathepsin D is associated with liver fibrosis [108]. Cathepsin B expression in the context of macrophage pyroptosis and systemic sclerosis is also associated with increased fibrosis [109]. Here, we consider how lysosomal cathepsin function contributes directly to intracellular collagen degradation.

The synthesis of cathepsins begins with the formation of the pre‐pro‐cathepsin in the ER. The nascent protein undergoes cleavage in the ER lumen by a signal peptidase to form a pro‐cathepsin. These pro‐cathepsins are further modified in the Golgi by the modification of mannose residues. Within lysosomes, pro‐peptide cleavage occurs in order to transform inactive pro‐cathepsins into active cathepsins. Activation can occur via two separate processes. Auto‐activation involves optimization of lysosomal pH and catalytic cleavage of cathepsin pro‐peptides, whereas trans‐activation involves separate lysosomal proteases [79].

Certain cathepsins are linked to the degradation of specific types of collagens. For example, cathepsin K targets Type I and II collagen [110] and requires glycosaminoglycans to cleave triple helical collagen [77]. Cathepsin L preferentially cleaves Type I collagen, similar to cathepsin K, albeit with lower collagenolytic activity, and produces fewer collagen fragments [111]. Cathepsin D targets Type I and III collagens [112] and can mediate the maturation of procollagen by removing the COOH‐terminal pro‐peptide to form mature collagen [113]. On the other hand, cathepsin S cleaves elastin (a major ECM fibrillar protein) and regulates the production of Type IV collagen [114]. Because of the importance of lysosomal pH in cathepsin function [79], we consider below how lysosomal pH is acidified to optimize collagen degradation.

## 14 Function of Vacuolar H^+^‐ATPases (V‐ATPases) in Collagen Degradation

The acidic pH of lysosomes is created in part by the functional activities of V‐ATPases [79], which are conserved proton pumps in mammalian cells that generate proton gradients used for cellular trafficking, degradation, and endocytosis [115]. When V‐ATPases are overexpressed in cells, lysosomal pH is reduced compared with knockdown cells [116], indicating their utility in vacuolar acidification. V‐ATPases are also critical for cathepsin activation [117], a process that involves the triggering of autoproteolysis and activation of the enzyme [117, 118]. In addition, the low pH of lysosomes promotes conformational changes of substrates that optimize efficient degradation by cathepsins [79]. V‐ATPases help to mediate collagen degradation, as shown by experiments in which inhibition of V‐ATPase function decreases collagen degradation (up to 50% reduction of internalized collagen) [119].

Structurally, V‐ATPases contain an integral membrane domain (V0) that translocates protons across membranes using energy released from ATP hydrolysis, which is generated by the cytosolic V1 domain [115, 120]. The V‐ATPase B2 subunit, which is one of the subunits in the V1 domain involved in ATP hydrolysis, plays an important role in regulating lysosome function in response to oxidative stress [116]. In addition, V‐ATPases regulate and direct the transport of misfolded proteins for ER‐phagy [121].

The assembly of V‐ATPases and their generation of acidic environments are regulated in part by Ca^2+^ flux through ion channels embedded in lysosomal membranes. One particularly important ion channel is the transient receptor potential mucolipin 1 (TRPML1), a channel that is localized to late endosomes and lysosomes [122, 123] and conducts Ca^2+^ to enhance lysosomal degradation of proteins [122]. Ca^2+^ influx mediated by TRPML1 promotes the enlargement of lysosomes and vacuolar compartments, which are crucial in lysosomal biogenesis and function [123]. Alterations of Ca^2+^ permeability may affect intracellular lysosomal degradation and play a role in aggravating gingival enlargement.

## 15 Involvement of Rabs in Trafficking and Regulation of Ca^2+^ Channels

One of the most important systems for regulating trafficking of proteins through vacuolar compartments is the Ras‐associated binding (Rab) protein family (~60 different proteins). Rabs are small GTPases that localize to different types of vacuolar compartments to regulate vesicle formation, movement, tethering, and fusion [124]. Newly synthesized Rabs undergo post‐translational modifications (e.g., prenylation) to enable appropriate delivery to target membranes [125]. Like other small GTPases, Rabs cycle between an active state (GTP‐loaded) and an inactive state (GDP‐loaded) as a result of the activity of guanine nucleotide exchange factors and GTPase‐activating proteins, respectively. Active GTP‐bound Rabs mediate effector functions as a result of binding to target membranes. For example, enrichment and activation of Rab7 in the membrane of late endosomes and lysosomes promote intracellular internalization via endocytosis and autophagy, respectively [126].

As described above, various drugs (Table 1) dysregulate Ca^2+^ signaling and inhibit intracellular collagen degradation by poorly described mechanisms. Notably, Ca^2+^ flux through certain Transient Receptor Potential ion channels is regulated by specific Rabs. For example, trafficking and activation of the Ca^2+^ permeable channel TRPC6 is governed by Rabs 9 and 11 [127]. The Cystic Fibrosis Transmembrane Conductance Regulator (CFTR) contributes to the flux of Cl^−^ and other ions across cell membranes in epithelial cells and also plays an important role in lysosomal degradation. The endocytosis and localization of CFTR are regulated by Rab5 and Rab7, respectively, whereas trafficking of the CFTR to the trans‐Golgi Network is affected by Rab11 [128], which also regulates Ca^2+^ transport by binding to TRPV6 channels. In addition, trafficking of Orai1, a protein that is a critical functional element of Ca^2+^ Release‐Activated Ca^2+^ (CRAC) channels and that enables store‐operated Ca^2+^ entry, is also regulated by Rab5 (at early endosomes) and Rab7 (at late endosomes) [129]. Taken together, certain Rabs evidently play diverse regulatory roles in permeation of Ca^2+^ through ion channels, underlining their linkage to Ca^2+^ signaling and their impact on lysosomal degradation.

## 16 Role of Rabs in Regulation of Collagen Homeostasis

**FIGURE 3:**
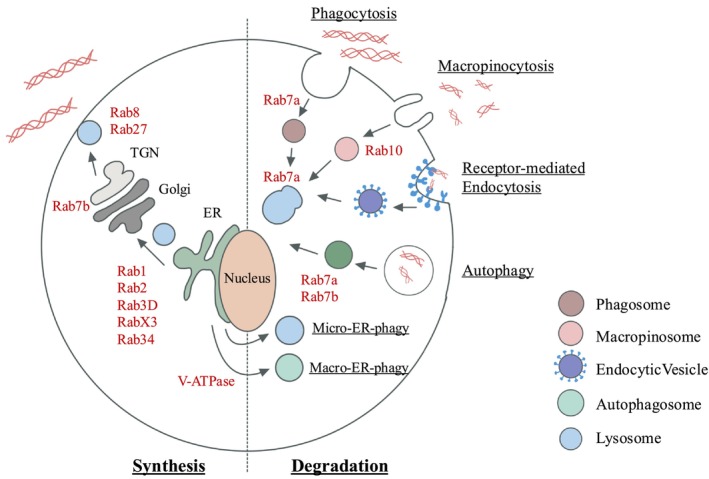
Overview of vacuolar systems involved in collagen homeostasis.

In addition to their enabling of lysosomal maturation and function, Rab family members also regulate collagen synthesis, which underlines their critical roles in collagen homeostasis. Rabs affect the trafficking of vacuoles to govern fusion of phagosomes and lysosomes, which enables the formation of phagolysosomes, one of the compartments in which collagen is degraded [130, 131]. Because of the central role of Rabs in vacuolar trafficking, we consider below their contributions to tuning the rate and extent of collagen synthesis and degradation (Figure 3), a fruitful area of matrix biology research.

## 17 Involvement of Rabs in Collagen Synthesis and Degradation

For collagen synthesis, and specifically in the procollagen glycosylation step, Rab1 and Rab3D are required for trafficking procollagen from the ER to the Golgi. Rab27a subsequently traffics the nascent protein to the plasma membrane for export [132]. As described above, collagen transport from the ER requires ER exit sites and TANGO1 proteins [133]. For this process, Rab1 is concentrated in the TANGO1 ring to regulate collagen secretion and transportation from the ER at ER exit sites [134]. Subsequently, Rab34 is involved in trafficking collagen to the Golgi prior to secretion [135].

Extracellular degradation of collagen also involves Rabs for the transport of molecules required for collagenolysis. For example, Rab8 is required for the transport of MT‐1 MMP towards the plasma membrane [136]. There, MT1‐MMP is secreted in its catalytically inactive form. When activated by furin, a proprotein convertase, MT‐1 is transported across the plasma membrane, where it can partially degrade extracellular collagen prior to internalization by micropinocytosis [77]. This process is regulated by Rab10 [137], which is phosphorylated by leucine‐rich repeat kinase 2 to drive the formation, maturation, and recycling of macropinosomes [137, 138].

**FIGURE 4:**
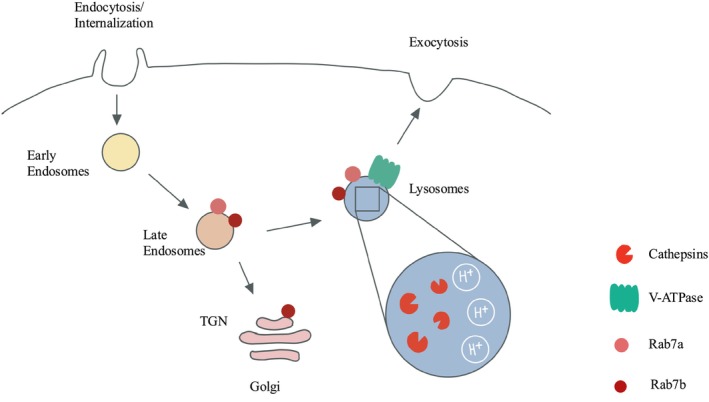
Role of Rabs and V‐ATPases in lysosomal trafficking and degradation.

Not all of the functions of the ~60 different Rabs are well‐defined; some of these proteins may exhibit overlapping functions [139]. When considering a role for Rabs in the control of vacuolar traffic to enable intracellular collagen degradation, convincing data demonstrate that Rab7 is involved in the trafficking of vacuoles involved in protein degradation [140]. There are two paralogues of Rab7: Rab7a and Rab7b [141]. Rab7a localizes to late endosomes and lysosomes, where it regulates early to late endosomal maturation and late endosome to lysosome maturation. Rab7b is involved in trafficking cargoes between endosomes and the Golgi and localizes to the Trans Golgi Network. Interactions between Rab7a and its effector, Rab7‐interacting lysosomal protein, can regulate V‐ATPase‐mediated acidification of lysosomes (Figure 4) [141]. In addition, Rab7a can govern MMP‐2 activity, which is involved in the extracellular degradation of collagen [142]. Further, Rab7a determines the localization and activation of the β1 integrin (involved in the initial binding of collagen to cells) and the formation of cell protrusions known as filopodia and cell spreading [143]. This involvement of Rab7a in regulating β1 integrin function suggests that this Rab affects early events in collagen degradation, in which cells initially bind to collagen, a rate‐limiting step in the intracellular pathway.

The intracellular collagen degradation pathways involved in phagocytosis, macropinocytosis, and receptor‐mediated endocytosis all converge on the fusion of phagosomes with lysosomes, a process that is mediated by Rab7a [144]. In addition, autophagy, another pathway for the degradation of internalized collagen [145], is differentially regulated by Rab7a and Rab7b [146]: Rab7a upregulates and Rab7b downregulates autophagy, respectively [132]. Rab7b regulates cathepsin D maturation through its transport from the Trans Golgi Network to endosomes and lysosomes [147]. Depleting Rab7b increases the abundance of pro‐cathepsin D, the immature form of cathepsin D [147]. Collectively, these data indicate that Rabs likely play important roles in governing intracellular collagen degradation. Indeed, there is increased interest in Rabs as target proteins for treating fibrotic lesions [148, 149, 150].

## 18 Interaction of V‐ATPase With Rabs for Lysosomal Enrichment

The activity of V‐ATPases is controlled by their localization to endomembranes, such as lysosomes. This process is mediated by the small *a* subunit of V‐ATPases [151]. In mammals, there are four *a* isoforms (a1–4); *a*3 is enriched in late endosomes and lysosomes [151]. The *a*3 subunit interacts with inactive forms of the small GTPases Rab7 and Rab27a [151], which localize these Rabs to vacuoles (i.e., lysosomes) that are involved in collagen degradation. The V‐ATPase *a*3 subunit also regulates the localization of Mon1‐Ccz1, a guanine nucleotide exchange factor that activates Rab7 associated with lysosomes [152].

## 19 Applying Insights on Rabs for Regulation of Intracellular Collagen Degradation

**FIGURE 5:**
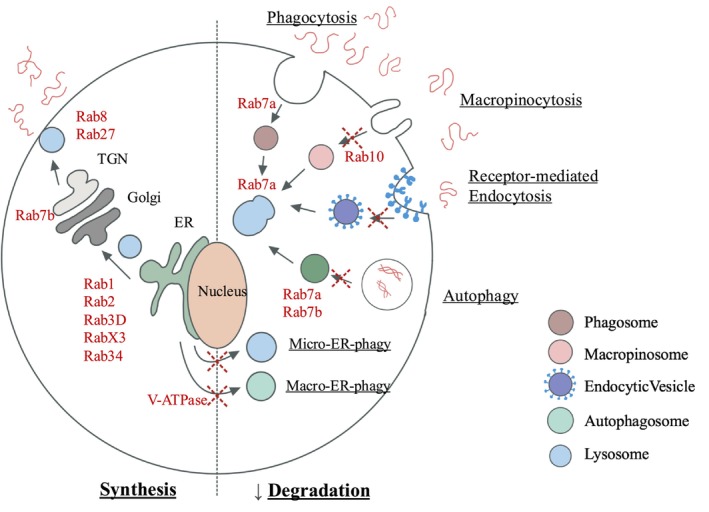
Potential disruption of vacuolar systems in gingival fibroblasts that impacts intracellular degradation of collagen. The figure illustrates how different Rabs affect specific vacuolar systems in cells that may contribute to the internalization and intracellular degradation of collagen. Extracellular collagen is shown as brown‐colored “squiggles” in the intracellular space. Collagen is trafficked through discrete internalization systems that ultimately deliver collagen to Rab7a compartments for digestion by lysosomal hydrolases such as cathepsins.

In spite of a detailed understanding of how the extracellular collagen degradation pathway is regulated, targeting MMPs to improve ECM homeostasis has not led to marked improvements in human health [153]. The lack of large increases in collagen synthesis in gingival enlargement suggests that impaired intracellular degradation of collagen is likely responsible for the excess extracellular collagen in fibrotic gingiva. Conceivably, different gingival enlargement‐promoting drugs may affect intracellular degradation in discrete ways. For example, the deposition of misfolded collagen could cause structural changes and inappropriate assembly of collagen that blocks its internalization and degradation. Further, if MMPs in the extracellular pathway cannot target and partially degrade misfolded collagen, the absence of cleaved fragments may affect collagen internalization by macropinocytosis or receptor‐mediated endocytosis. Misfolded collagen may also affect ligand binding in receptor‐mediated endocytosis. Accordingly, perturbations of the intracellular degradation pathway may be of particular importance in gingival enlargement (Figure 5).

## 20 Current Challenges

Several experimental methods have been developed to label collagen for studies of collagen internalization, including, for example, electron microscopic study of banded collagen fibrils in vacuoles [154], intratumoral injections of fluorescently labeled collagen [155] and incubation of cells with labeled matrix molecules [156]. Injecting labeled collagen in animal models enables visualization of collagen internalization [155] but does not easily resolve which internalization pathway is used in vivo. New technologies are needed that can simultaneously measure internalization kinetics and define the vacuolar compartments through which the collagen is trafficked.

Because of the tight helical structure of collagen, attaching and preserving a fluorescent label on native fibrillar collagen while maintaining normal biological functions and structural features is challenging [157]. As intact collagen is internalized through phagocytosis [83], labeling exogenous collagen to enable vacuolar‐specific location and integrin‐specific binding could provide new insights into control of the phagocytic route [158]. Examination of internalization pathways with labeled soluble collagen fragments and fluid‐phase markers of macropinosomes [159] and tagging clathrin‐coated vesicles [160] could be used to distinguish macropinocytosis and receptor‐mediated endocytosis pathways.

As separate regulatory systems likely govern the kinetics of collagen internalization and degradation, identifying the impact of pro‐fibrotic drugs on specific regulatory systems will be challenging. Further, as collagen‐binding molecules like decorin affect collagen phagocytosis [161], a more complete understanding of these processes may need an improved definition of the macromolecular complexes that are being internalized.

## 21 Future Perspectives

Detailed analyses of how critical regulatory molecules in the intracellular pathway (such as Rabs) impact collagen remodeling could lead to better clinical management of ECM health, particularly in gingival enlargement. For example, defining how specific Rabs interact with and regulate endomembrane vacuolar systems and subcellular trafficking of collagen degradation could provide insights into which specific drugs that cause gingival enlargement affect certain subpopulations of patients but not others. It will be important to determine whether Rabs regulate membrane‐anchored MMPs (e.g., MT‐1) and cathepsins, and whether these processes are affected in patients with gingival enlargement. Further, understanding how dysfunction of Rabs and V‐ATPases contributes to gingival enlargement could inform how these molecules are perturbed in the fibrosis of other organs. As lung, skin, and liver fibrosis are reported to involve dysregulated collagen degradation pathways [82], targeting potential vacuolar regulators of collagen degradation may provide insight into the clinical management of fibrosis.

As described above in the section on inflammation‐associated gingival enlargement, which likely includes TGF‐β and related networks to increase collagen synthesis, we note that inflammatory signaling can modulate the activity of Rabs and V‐ATPases, which may suggest pathogenic mechanisms that reflect clinical phenotypes (i.e., drugs ↓collagen degradation + inflammation ↑collagen synthesis → ECM accumulation).

Currently, it is not known whether gingival enlargement is affected by regulatory pathways involved in collagen degradation, such as cAMP. However, recent data on lung fibroblasts indicate that the acidification of lysosomes and collagenolytic activity are enhanced through the activation of a G‐protein coupled receptor, dopamine receptor D1, that promotes increased cAMP activity [162]. Further, Traf2 and NCK Interacting Kinase regulates collagen degradation when there are high levels of procollagen I in cells [163]. Defining the key regulators of collagen degradation may provide additional insight into the pharmacology of the drugs that cause gingival enlargement.

## Author Contributions

W.H.W. and C.A.M. conceived, organized, wrote, and edited all versions of the manuscript. W.H.W. composed the figures and tables. R.A.Z. and M.F.M. revised the final version of the manuscript.

## Conflicts of Interest

The authors declare no conflicts of interest.

## Data Availability

The authors have nothing to report.
